# Antifungal susceptibility of *Aspergillus* spp. isolated from coffee beans of Los Santos Coffee Region, Costa Rica

**DOI:** 10.3389/fpubh.2026.1817727

**Published:** 2026-05-20

**Authors:** Daniela Jaikel-Víquez, Mariana Sancho-Chaverri, Víctor Gabriel Villegas-Ramírez, Ian Cambronero-Ortiz, Isaac Santamaría-Sánchez, Fernando Morales-Calvo, Sebastián Rodriguez Saravia, Alejandra Calderón-Hernández, Norma T. Gross

**Affiliations:** 1 Mycology Section, Department of Microbiology and Immunology, School of Microbiology, Universidad de Costa Rica, San Pedro, San José, Costa Rica; 2 Centro de Investigación en Enfermedades Tropicales (CIET), Universidad de Costa Rica, San Pedro, San José, Costa Rica; 3 Clinical Laboratory, Hospital Nacional de Geriatría y Gerontología Dr. Raúl Blanco Cervantes, Caja Costarricense del Seguro Social, San José, Costa Rica; 4 Centro de Investigación en Contaminación Ambiental (CICA), Universidad de Costa Rica, San Pedro, Costa Rica; 5 Mycology Laboratory, School of Veterinary Medicine, Universidad Nacional, Heredia, Costa Rica

**Keywords:** amphotericin B, antimicrobial resistance, *Aspergillus*, coffee, itraconazole, voriconazole, One Health

## Abstract

The indiscriminate use of fungicides in agricultural crops is considered one of the most important causes of antifungal resistance. Hence, it is our interest to contribute to the knowledge of antifungal resistance, within the framework of One Health. The results will be useful to define appropriate policies in the use of these drugs both in the clinical area and in agriculture. Thus, the susceptibility patterns of 187 *Aspergillus* spp. (*A. flavus* [*n* = 46], *A. fumigatus* [*n* = 8], *A. insuetus* [*n* = 1]; *A. niger* [*n* = 85], *A. tamarii* [*n* = 34] and *Aspergillus* section *Versicolores* [*n* = 13]), isolated from coffee beans from the Los Santos coffee-growing region in Costa Rica, were evaluated for amphotericin B (AMB), itraconazole (ITZ) and voriconazole (VRC); using the Clinical and Laboratory Standards Institute (CLSI) broth microdilution M38 guideline. The mean minimal inhibitory concentration (MIC) for AMB was 2.77 ± 3.19 μg mL^−1^, 0.29 ± 0.26 μg mL^−1^ for ITZ, and 1.14 ± 2.68 μg mL^−1^ for VRZ. Based on the CLSI guidelines, 15.2% of *A. flavus*, 75.0% of *A. fumigatus* and 3.5% of the *A. niger* were classified as non-wild type for AMB; 4.3% of the *A. flavus/oryzae* and 25.0% of the *A. fumigatus* were classified as non-wild type for VRC. ITZ presented the lowest MIC distribution, and the isolates were classified as wild-type strains. The results show a high susceptibility to ITZ, moderate to VRC (treatment of choice for invasive aspergillosis) and low susceptibility to AMB.

## 1 Introduction

Aspergillosis is one of the most prevalent invasive fungal diseases that has a high morbidity and mortality rate in patients with some degree of immunosuppression. This disease has been reported in patients with neutropenia, solid organ transplant recipients, debilitating diseases, systemic use of glucocorticoids, treatment with T-cell immunosuppressants, inherited immunodeficiencies (1–3), chronic lung disease, severe viral respiratory infections and patients in critical condition due to COVID-19 (4). The three most important conditions of the clinical spectrum of pulmonary aspergillosis are: allergic bronchopulmonary aspergillosis, chronic pulmonary aspergillosis and the most serious one, invasive pulmonary aspergillosis (5). *Aspergillus* spp. are responsible for more than 200,000 cases of invasive aspergillosis per year, more than 1.2 million cases of chronic pulmonary aspergillosis and 10 million cases of severe asthma with fungal sensitization (6, 7). They are also causative agents of superficial illnesses like onychomycosis, otomycosis and keratitis (8). Among the species that have been linked to human disease, *Aspergillus fumigatus* causes the majority of cases ([75–85] %), followed by *Aspergillus flavus* ([5–10] %), *Aspergillus niger* ([1.5–3] %), and *Aspergillus terreus* ([2–3] %) (9).

For the treatment of aspergillosis, azoles are recommended, with voriconazole (VRC) being the antifungal of choice. It is important for patients to receive treatment since mortality rates for patients with invasive aspergillosis that are not medicated are (80–95) % (10). However, despite advances in antifungal therapy, resistance to antifungals has become a growing problem worldwide. Ever since the first case of azole-resistant *A. fumigatus* was reported in 1989, in the state of California, United States of America (11), and other cases were documented in the late 1990s in the United Kingdom and Sweden; the increasing azole-resistance has made it difficult to treat invasive pulmonary aspergillosis (12).

The overall prevalence of azole resistance is estimated to be between three and 6 %. In addition, resistance has been found in patients who had no previous exposure to antifungals (13). It is worth noting that resistance has been documented after the indiscriminate use of triazole fungicides in agricultural products, which are chemically similar to antifungals used in clinical treatments (14–16). For example, Rhodes et al. (15) published a paper on population genomics confirming that the acquisition of drug-resistant *A. fumigatus* infections by humans came from the environment. The route of acquisition of environmental resistance is one of the most studied and the one that generates the greatest concern in the medical community. These fungicides can persist in the environment for many weeks and have been reported to travel; thus, exposure and selection should be considered beyond the sites of application at agricultural fields (16). Consequently *Aspergillus* spp. can initiate adaptation strategies to environmental stress that generate mutations that make them resistant to triazoles for agricultural and medical use (17). Mutations in the *CYP51A* gene (TR34/L98H and TR46/Y121F/T289A) are the most frequently found in resistant strains, while 30% are related to changes in effluent pumps, leading to therapeutic failure and a 90% increase in mortality (18).

Coffee is one of the most important crops grown in Costa Rica. Unfortunately, it can get infected by *Hemileia vastatrix* (coffee leaf rust) and mycotoxigenic fungi, including members of the genus *Aspergillus* (19). Thus, different azole-based compounds are applied to prevent said infections: cyproconazole, epoxiconazole, propiconazole and tebuconazole, for example (20), which could induce secondary resistance to the azoles approved for human treatment. It is important to emphasize that the Ministry of Agriculture and Livestock of Costa Rica (MAG, from the Spanish *Ministerio de Agricultura y Ganadería*) estimated an importation of (9864–12,573) tons per year of pesticides with an apparent use of (4441–9,209) tons per year, between 2017 and 2023; out of which, 61.90% were fungicides (21). Hence, it is our interest to contribute to the knowledge of antifungal resistance, within the framework of One Health. This term is often defined as “an integrated, unifying approach that aims to sustainably balance and optimize the health of people, animals, and ecosystems. It recognizes the health of humans, domestic and wild animals, plants, and the wider environment (including ecosystems) are closely linked and interdependent” (22). Therefore, since, chronic exposure to azolic plaguicides has been previously reported to select resistant strains; we wanted to monitor the susceptibility patterns of environmental *Aspergillus* spp. isolated from Costa Rican coffee beans. As it is of great importance to know about the situation of resistance to antifungals, for it will allow treating physicians to make an appropriate decision. This knowledge will lay the foundation for a better response to patients with invasive aspergillosis, thereby reducing mortality and medical costs.

## 2 Materials and methods

### 2.1 *Aspergillus* spp. isolates

The minimal inhibitory concentration (MIC) was determined for 187 *Aspergillus* spp. obtained from a previous research study (19). In summary, the isolates were obtained from cherry, green and roasted coffee beans from a cooperative that operated a coffee processing facility in the largest coffee region of Costa Rica, the Los Santos coffee- growing area. The sample size was determined by using the Cochran formula and the samples were obtained randomly from two harvests: 2019–2020 and 2020–2021. In said study (19) the fungal isolates were identified by mass spectrometry using the Maldi Biotyper® (Bruker Daltonics, Billerica, MA, USA). The spectra were analyzed using the Bruker Library and the MSI (Mass Spectra Identification; https://msi.happy-dev.fr) platform developed by the Assistance Publique-Hôpitaux of Paris, the Sorbonne University of Paris and the Belgian Coordinated Collections of Microorganisms (BCCM). They were classified as *Aspergillus flavus/oryzae* (*n* = 46), *Aspergillus fumigatus* (*n* = 8), *Aspergillus insuetus* (*n* = 1); *Aspergillus niger* aggregate (*n* = 85), *Aspergillus tamarii* (n = 34) and *Aspergillus* section *Versicolores* (*n* = 13) and were deposited in the Fungal Collection of the School of Microbiology of the Universidad de Costa Rica (VI-B7732) and maintained in test tubes with potato dextrose agar (PDA) and Czapek Dox agar, at room temperature (ca. [20–25] °C).

### 2.2 Inoculum preparation

Each *Aspergillus* isolate was subcultured in test tubes with PDA and incubated for 7 days at room temperature. Subsequently, conidia suspensions were prepared in sterile 0.85% saline solution and their concentration was standardized to (1–5) × 10^6^ conidia mL^−1^, using a Neubauer counting chamber (Hausser Scientific, Horsham, PA, United States of America). Two hundred microliters of each suspension were added to test tubes with 9.8 mL of Roswell Park Memorial Institute (RPMI) 1640 broth (Thermo Fisher Scientific, United States of America). Control strains from the American Type Culture Collection (ATCC) were used as controls of the antifungal medium and concentrations tested: *Candida krusei* ATCC 6258 and *Candida parapsilosis* ATCC 22019. For the inoculum of the control strains, a 24-h subculture in Sabouraud’s dextrose agar of the yeasts was used. From these colonies, blastospore suspensions were made to a 0.5 McFarland optical density in sterile 0.85% saline solution to obtain a concentration of approximately (1–5) x 10^6^ blastospores mL^−1^. Finally, they were diluted to 1:1 000 with RPMI 1640 to obtain a final concentration of (1–5) x 10^3^ blastospores mL^−1^.

### 2.3 *In vitro* susceptibility testing for the antifungals itraconazole, voriconazole, and amphotericin B

The determination of the MICs was carried out as specified in the M38 document: “Reference Method for Broth Dilution Antifungal Susceptibility Testing of Filamentous Fungi” third edition (23). Stock solutions of the antifungals itraconazole (ITZ), voriconazole (VRC) (Royal Pharm, Hangzhou, China), and amphotericin B (AMB) (Sigma Chemicals Co., St. Louis, Mo, USA) were prepared at a concentration of 1,600 μg mL^−1^ using dimethyl sulfoxide (DMSO) (Sigma Chemicals Co., St. Louis, Mo, United States of America) as a diluent. From the stock solutions, dilutions were prepared at concentrations one hundred times higher than the final concentrations tested and then diluted 1:50 with RPMI 1640 broth, to obtain concentrations twice as high as the final concentration. The final concentrations tested were: (0.03–16.00) μg mL^−1^.

Microtiter plates were filled with 100 μL of each antifungal dilution and inoculated with 100 μL of the spore suspension. Growth and reagent controls were prepared by adding 100 μL of RPMI 1640 medium with DMSO at a concentration of 1%. Then, the growth control was inoculated with 100 μL of the spore suspension and 100 μL of RPMI 1640 were added to the reagent control well. All tests were performed by duplicate.

The results were read visually, after incubation of the plates for 48 h at 30 °C and without agitation. The MIC was defined as the lowest concentration of antifungal that completely inhibited fungal growth when compared to the growth control.

### 2.4 Statistical analysis

The range, geometric mean with standard deviation, and 50th (MIC_50_) and 90th (MIC_90_) percentiles of the MIC data were calculated for every antifungal tested, for every *Aspergillus* species and for all the isolates together. The normality of the MIC distributions was evaluated with Kolmogorov–Smirnov test for *Aspergillus* spp. (*n* = 187) and *A. niger* aggregate (*n* = 85) and with Shapiro–Wilk test for *A. flavus/oryzae* (*n* = 46), *A. tamarii* (*n* = 34), *Aspergillus* section *Versicolores* (*n* = 13) and *A. fumigatus* (*n* = 8). Then, statistical differences between the MICs obtained for each antifungal, for each specie were evaluated using a Kruskal-Wallis test with a Dunn’s *Post Hoc* test (*p* < 0.05). The statistical analysis was performed with the SPSS program, version 20 (SPSS Inc., Chicago, III, United States of America).

### 2.5 Minimal inhibitory concentration interpretation

MIC interpretations were obtained using the M57S document: “Epidemiological Cutoff Values for Antifungal Susceptibility Testing,” 4th edition (24). The epidemiological cutoff values (ECV) available for AMB, ITZ and VRC, respectively, are: *A. flavus* 4 μg mL^−1^, 1 μg mL^−1^ and 2 μg mL^−1^; *A. fumigatus* 2 μg mL^−1^, 1 μg mL^−1^ and 1 μg mL^−1^ and *A. niger* 2 μg mL^−1^, 4 μg mL^−1^ and 2 μg mL^−1^.

## 3 Results

**Figure 1:**
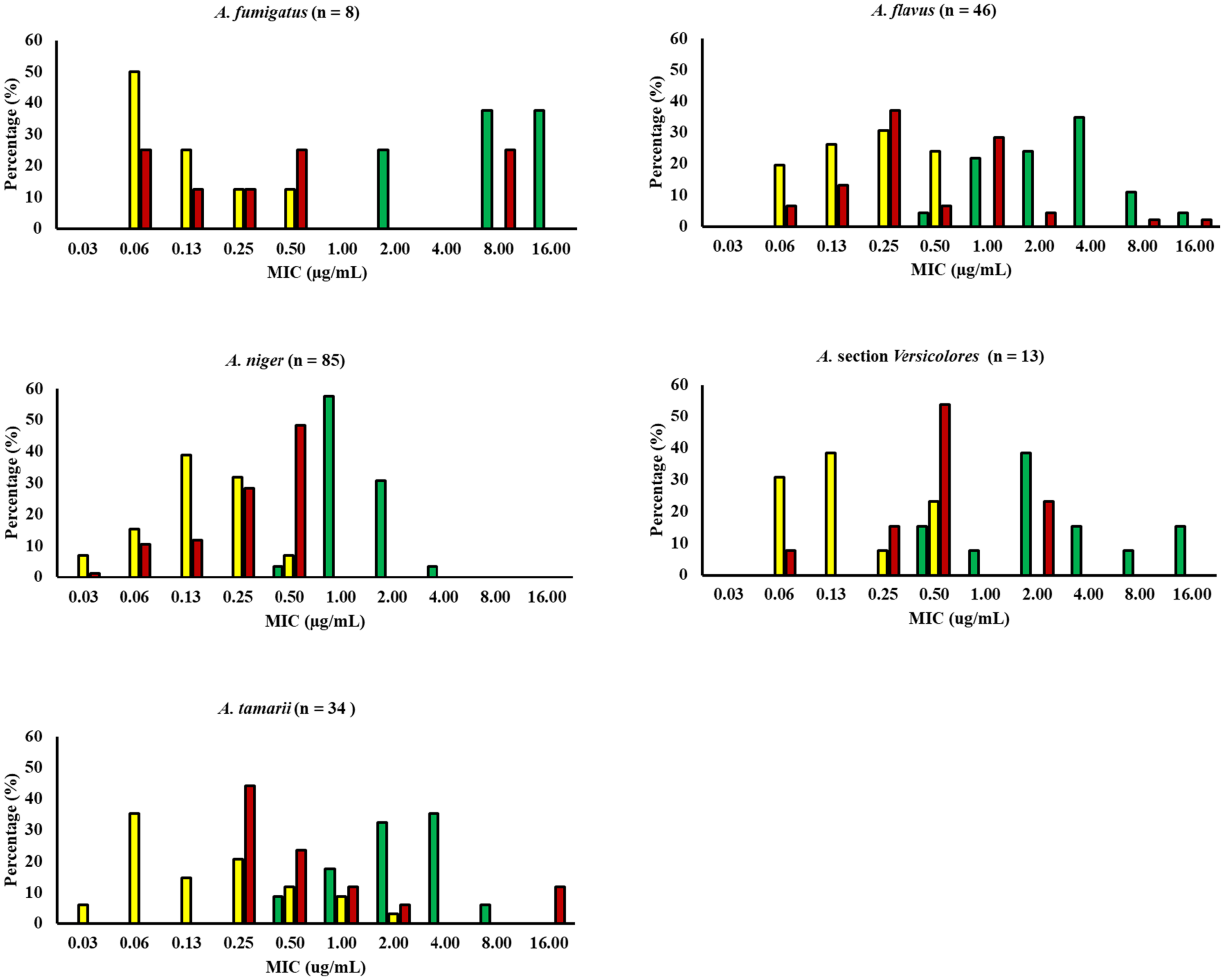
Minimal inhibitory concentration (MIC) distribution of *Aspergillus* spp. isolated from coffee beans from Los Santos Coffee Region in Costa Rica. The yellow bars represent itraconazole, the red bars voriconazole, and the green bars amphotericin B.

The antifungal susceptibility patterns to AMB, ITZ and VRC of the n = 187 *Aspergillus* spp. were determined by the CLSI M38 guideline. The mean MIC (for all isolates) for AMB was 2.77 ± 3.19 μg mL^−1^, 0.29 ± 0.26 μg mL^−1^for ITZ, and 1.13 ± 2.68 μg mL^−1^ for VRC. The MIC distributions for each species are presented in Table 1 and Figure 1. Statistical differences were found between the MICs obtained for each antifungal agent. In the case of all the isolates and *A. niger*, the MICs obtained for the three antifungal agents were statistically different from each other (*p* < 0.001). In the case of the other species, statistical differences were also found (*p* < 0.05), but the *post Hoc* test grouped ITZ and VRC together and AMB in a different group.

**Table 1:** Susceptibility patterns of *Aspergillus* spp. isolated from coffee beans from Los Santos Coffee Region in Costa Rica (*n* = 187).

Species	Number	Antifungal agent	MIC range	MIC_50_	MIC_90_	Mean ± SD
*A. flavus/oryzae*	*n* = 46	AMB	0.50–16.00	3.00	8.00	3.67 ± 3.41
		ITZ	0.06–0.50	0.25	0.50	0.24 ± 0.16
		VRC	0.6–16.00	0.25	1.30	1.04 ± 2.55
*A. fumigatus*	*n* = 8	AMB	2.00–16.00	8.00	NA	9.50 ± 5.93
		ITZ	0.06–0.50	0.10	NA	0.16 ± 0.15
		VRC	0.60–8.00	0.38	NA	2.19 ± 3.59
*A. insuetus*	*n* = 1	AMB	NA	NA	NA	4.00
		ITZ	NA	NA	NA	0.25
		VRC	NA	NA	NA	0.50
*A. niger* aggregate	*n* = 85	AMB	0.06–4.00	1.00	2.00	1.35 ± 0.75
		ITZ	0.06–1.00	0.25	0.50	0.35 ± 0.24
		VRC	0.06–2.00	0.50	1.00	0.67 ± 0.37
*A. tamarii*	*n* = 34	AMB	0.50–8.00	2.00	4.00	2.75 ± 1.86
		ITZ	0.03–2.00	0.13	1.00	0.30 ± 0.41
		VRC	0.03–16.00	0.50	16.00	2.35 ± 5.08
*Aspergillus* section *Versicolores*	*n* = 13	AMB	0.50–16.00	2.00	16.00	4.61 ± 5.42
		ITZ	0.06–0.50	0.13	0.50	0.20 ± 0.18
		VRC	0.06–2.00	0.50	2.00	0.77 ± 0.71

NA, Not applicable; AMB, amphotericin B; ITZ, itraconazole; VRC, voriconazole.

**Figure 2:**
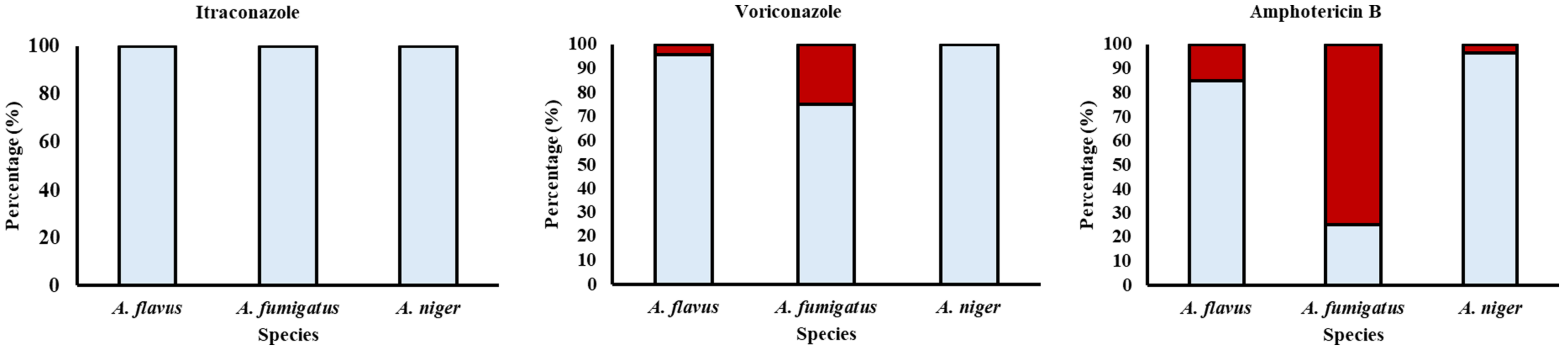
Classification of *Aspergillus* spp. isolated from coffee beans from Los Santos Coffee Region in Costa Rica in wild types (light blue) and non-wild types (red) according to the environmental cut-off values.

When comparing the MICs to the ECVs, for AMB 15.2% (*n* = 7) of the *A. flavus/oryzae*, 75.0% (*n* = 6) of *A. fumigatus* and 3.5% (*n* = 3) of the *A. niger* aggregate were classified as non-wild types. Regarding VRC, 4.3% (*n* = 2) of the *A. flavus/oryzae* and 25.0% (*n* = 2) of the *A. fumigatus* were classified as non-wild type for VRC. As for ITZ, the isolates analyzed of the three species were all classified as wild-types (Figure 2). It is worth mentioning that ECVs are different from clinical breakpoints. The ECV is the MIC that separates microbial populations into those with and without acquired and /or mutational resistance. They are obtained from analyzing MIC distributions of a large number of isolates tested using a susceptibility testing method, while clinical breakpoints come from MIC distribution, pharmacokinetic and pharmacodynamic data and clinical efficacy data. Therefore, ECVs are not predictors of clinical success of failure (25).

## 4 Discussion

In Costa Rica, azole-based compounds are used in coffee fields to avoid fungal infection of the plants. As previously stated, the azoles used are cyproconazole, epoxiconazole, propiconazole and tebuconazole (20). These fungicides are short chained triazoles which are used for their broad antifungal spectrum (26–29). Unfortunately, patients with aspergillosis are also treated with azoles: VRC, ITZ, posaconazole and isavuconazole (2, 30), being the first a short-chained azole and the second a long-chained azole (31, 32). Since aspergillosis is a community obtained disease, the exposure to fungal isolates with secondary resistance to azoles is of concern. Therefore, the present study, determined the susceptibility patterns of 187 *Aspergillus* spp. obtained from coffee beans from the Los Santos Coffee Region in Costa Rica. It is worth noting that *in vitro* antifungal profiles for environmental *Aspergillus* are scarce.

Our results showed a higher susceptibility to ITZ, followed by VRC and lastly by AMB. In the case of the azole-based drugs, the MICs obtained for ITZ were lower than those reported in Taiwan (33), Portugal (34), China (2) and Iran (35). In the case of VRC, the MIC values were similar to those from other studies (2, 33, 36–39). Also, it is important to highlight that the mean MICs obtained for the Costa Rican environmental *A. fumigatus*, for both azoles, were like those reported from clinical isolates from Costa Rica (40).

Azoles act by inhibiting the enzyme 1,4–*α*–lanosterol–demethylase, which alters ergosterol biosynthesis and thus alters cell membrane functionality, ultimately leading to cell death or inhibition of growth and replication (41). Among the known mechanisms of resistance are mutations resulting in decreased affinity for azoles or overexpression of the *cyp51A* gene (2). It is worth mentioning that Chowdhary et al. (42) isolated *A. fumigatus* resistant to VRC from potato (*Solanum tuberosum*) fields and from fenugreek (*Trigonella foenum-graecum*) fields and ITZ resistant strains from rose (*Rosa* spp.) bed soil and red chilli (*Capsicum annuum*) fields from India. The authors reported that the VRC-resistant isolates exhibited the TR46/Y121F/T289A mutation and the ITZ-resistant isolates the TR34/L98H mutation (42). Ren et al. (43) also described these mutations in *A. fumigatus* strains isolated from soil samples from greenhouses that grow vegetables and fruits from Zhejiang, China; epoxiconazole, tebuconazole, propiconazole, hexaconazole and metconazole were used as fungicides in these crops. Therefore, the authors suggest a link between the agricultural use of triazoles and the appearance of resistance in *A. fumigatus* to triazole drugs used for treating human beings (43).

As an alternative therapy to first-line triazole treatments AMB is used for invasive aspergilosis. It is a polyene that acts by binding to ergosterol causing the formation of ion channels in the cell membrane and therefore the death of the fungus (41). In contrast to what we found for azoles, the MICs obtained for AMB were higher than those reported from studies performed with clinical isolates from Taiwan (33) and Portugal (34) but were similar to those obtained in Venezuela (38) and Iran (35). Even though resistance to AMB is rare in fungi, low susceptibility patterns have been reported in other *Aspergillus* spp. like *A. terreus*, which is even considered innately resistant to this drug (44, 45).

Low susceptibility was found for AMB, especially for *A. fumigatus.* However, it is important to emphasize that one of the limitations of this study is that only eight isolates of the latter species were analyzed. So, it is recommended to expand the sample size of environmental *A. fumigatus* maybe by including isolates from other foods. In the case of VRC MIC ranges were broadly distributed between the concentrations tested and the mean concentrations found for each species was higher than the one obtained for ITZ. In this regard, certain *A. flavus* and *A. fumigatus* strains showed a non-wild-type strains pattern to VRC, but not to IRZ. However, no statistical differences were found between the mean MICs of these two drugs. These findings highlight the importance of susceptibility testing to monitor clinical and environmental isolates and reaffirm the impact of pesticides used on the field on human health. Thus, it is recommended that more isolates of each species be tested and isolates from other crops where azole-based pesticides are used. Finally, a comparison between molecular analysis of possible mutations related to azole resistance found in environmental and clinical Costa Rican isolates would strength the linking of agricultural fungicides to resistance mechanisms.

## Data Availability

The original contributions presented in the study are included in the article/supplementary material, further inquiries can be directed to the corresponding author.
